# Severe acute respiratory distress syndrome in a woman infected with Ascaris lumbricoides

**DOI:** 10.2478/jccm-2025-0039

**Published:** 2025-10-31

**Authors:** Alexandra Elena Lazar, Mihai Claudiu Pui

**Affiliations:** George Emil Palade University of Medicine, Pharmacy, Science, and Technology of Targu Mures,Romania

**Keywords:** ARDS, Ascaris lumbricoides, parasitic infections, acute respiratory failure, helminth infections

## Abstract

Acute Respiratory Distress Syndrome [ARDS] is a critical condition characterized by severe respiratory failure due to widespread lung inflammation, which can arise from various causes including trauma, infections, and systemic diseases. Among the rare causes is infection with Ascaris lumbricoides, a helminth typically affecting the gastrointestinal tract but capable of causing severe respiratory complications. We present the case of a 41-year-old woman with acute respiratory distress and negative viral and bacterial tests, who was ultimately diagnosed with Ascaris lumbricoides-induced ARDS. Her management included mechanical ventilation, antimicrobial therapy, corticosteroids, and eventually anthelmintic treatment after discovering the parasite. Despite initial deterioration and severe hypoxemia, the patient improved significantly following anthelmintic therapy, allowing extubation on day 8 and ICU discharge on day 12. Helminth-induced ARDS, though rare, should be considered in critically ill patients, especially in endemic regions. Early identification and appropriate therapy can dramatically improve outcomes.

## Introduction

Acute Respiratory Distress Syndrome (ARDS) is a critical condition characterized by severe respiratory failure due to widespread lung inflammation, leading to increased alveolar-capillary membrane permeability. Its clinical signs and causes were described from the very beginning. This syndrome can arise from various etiologies, including infections, trauma, and other systemic diseases [1].

Among the less common causes of ARDS is parasitic infection, particularly by Ascaris lumbricoides. This helminth primarily affects the gastrointestinal tract but can lead to significant complications, including respiratory distress.

The association between Ascaris lumbricoides and ARDS is not widely recognized, making it an important area for clinical investigation and reporting. Ascaris lumbricoides is the largest intestinal nematode infecting humans and is prevalent in tropical and sub-tropical regions, where sanitation practices are often inadequate. It is estimated that approximately 891 million people worldwide are infected with this parasite, with a significant burden in endemic areas [2]. The clinical manifestations of Ascaris infection can range from asymptomatic to severe complications, particularly in cases of heavy worm burden. Complications may include intestinal obstruction, biliary obstruction, cholangitis, and even respiratory complications due to the migration of larvae through the lungs [3,4].

The larvae of A. lumbricoides can migrate to the lungs, causing a range of respiratory symptoms, which may culminate in ARDS, although this is a rare occurrence [5,6]. The pathophysiology of ARDS in the context of Ascaris infection may involve several mechanisms. The mechanical obstruction caused by adult worms can lead to localized inflammation and secondary infections, which may contribute to the development of ARDS [7]. Moreover, the immune response elicited by the presence of the parasite can lead to significant pulmonary inflammation, characterized by diffuse alveolar damage (DAD), which is a hallmark of ARDS [8].

In cases where the larvae migrate to the lungs, they can cause a hypersensitivity reaction, leading to further lung injury and respiratory distress [3,4].

The diagnosis of ARDS in the context of Ascaris infection can be challenging, as the clinical presentation may mimic other causes of respiratory failure. However, a thorough history, examination, appropriate imaging, and laboratory tests can aid in identifying the underlying cause. In cases where Ascaris is suspected, stool examination and imaging studies can reveal the presence of the parasite and associated complications, such as intestinal obstruction or biliary involvement [9,10].

## Case presentation

A 41-year-old woman from a rural area presented to the Emergency Department (ED) with acute respiratory distress, reporting progressive dyspnea over the preceding 4–5 hours and a 5-day history of fever and dry cough. On presentation, she exhibited severe respiratory distress, with a respiratory rate of 32 breaths per minute, oxygen saturation of 88% on room air, and a temperature of 38°C. She was hemodynamically stable with a blood pressure of 130/61 mmHg and a heart rate of 106 bpm in sinus rhythm. Her medical history was unremarkable, except for class I obesity (BMI = 27.68 kg/m^2^).

The patient reported self-medicating with acetaminophen (Perfalgan®, Bristol-Myers Squibb, France), cough suppressants, cefuroxime (*Cefuroxim Kabi®, Fresenius Kabi, Germany*), levofloxacin (Tavanic®, Sanofi-Aventis, France), metamizole (*Algocalmin®, Sanofi-Aventis, Romania*), and proton pump inhibitors during the symptomatic period before admission.

### Diagnostic Workup

Her respiratory condition rapidly worsened, requiring escalation to non-invasive ventilation (NIV). Due to persistent hypoxemia, she underwent emergency endotracheal intubation. Initial diagnostic investigations included routine laboratory tests, blood cultures, and tracheal aspirate cultures. A comprehensive respiratory viral panel, including SARS-CoV-2 and Influenza A/B, was also performed — all results were negative.

**Fig. 1.**
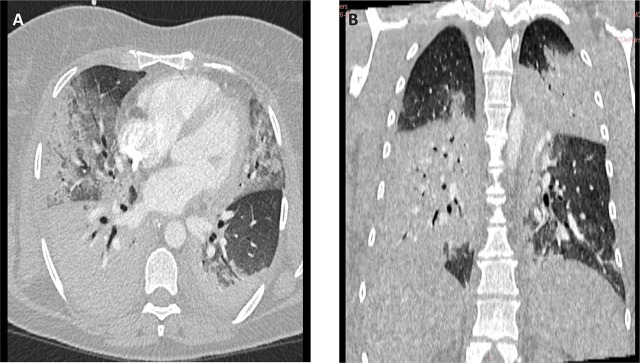
A. Thoracic CT angiography, axial section, acquired with intravenous contrast, pulmonary window. B. Thoracic CT angiography, coronal section, acquired with intravenous contrast, pulmonary window.

A thoracic CT angiography (CTA) revealed bilateral consolidations and ground-glass opacities consistent with severe pneumonia and ARDS (Figures 1A and 1B). Based on these findings, empirical broad-spectrum antibiotics such as vancomycin (Vancomycin Kabi®, Fresenius Kabi, Germany) and meropenem (*Meropenem Kabi®, Fresenius Kabi, Germany*) were initiated, alongside systemic corticosteroids like methylprednisolone 1 mg/kg/day ≈ 80 mg IV daily (*Solu-Medrol®, Pfizer, Belgium*) were administered, with tapering started after day 6 of ICU stay. Supportive therapies included intravenous crystalloids, aerosolized adrenaline, sedation with fentanyl (*Fentanyl Kalceks®, AS Kalceks, Latvia*) and midazolam (*Midazolam Kalceks®, AS Kalceks, Latvia*), and neuromuscular blockade with rocuronium bromide (*Esmeron®, MSD, Netherlands*) to facilitate mechanical ventilation.

### ICU Admission and Management

The patient was transferred to the Intensive Care Unit (ICU), where she was managed according to established ARDS protocols. Mechanical ventilation was performed using a lung-protective strategy, with tidal volumes of ≤6 mL/kg predicted body weight, and PEEP titrated as per lung mechanics and oxygenation target( with periodic prone positioning to optimize oxygenation. Empirical antimicrobial therapy was continued, alongside fluid resuscitation with crystalloids, anticoagulation with enoxaparin sodium (*Clexane®, Sanofi, France*), and nutritional support via a nasogastric tube. Additional therapies included mucolytics such as acetylcysteine *(Fluimucil®, Zambon, Italy*), gastric protection with proton pump inhibitors like pantoprazole (*Controloc®, Takeda GmbH, Germany*), and antipyretics (metamizole and acetaminophen) when the fever persisted.

Hemodynamic and respiratory monitoring – protective mechanical ventilation and prone positioning - was comprehensive, including continuous ECG, pulse oximetry, capnography, invasive blood pressure monitoring, central venous pressure measurement, and urine output tracking (Table 1).

**Table 1.** Paraclinical data

**DAY**	**1**	**2**	**3**	**4**	**5**	**6**	**7**	**8**	**9**	**10**	**11**	**12**	**Normal values**	**Units**
pH	7.31	7.45	7.50	7.43	7.43	7.39	7.66	7.53	7.55	7.63	7.37	-	7.35 7.45	-
PaO2	68	72	72	135	143	145	172	122	140	154	125	-	83 – 108	mmHg
PaCO2	72	51	46	51	50	64	29.0	42	33	26	43	-	35 – 45	mmHg
Lactate	0.8	2	1.2	1.4	1.6	1.5	1.5	1,4	2.2	1.3	1.3	-	<1.3	mmol/l
COHb	0.5	1.2	2.1	1	1.2	1.9	1.4	1	1.4	0.8	1.2	-	0.5–1.5	%
P/F	68	70	71	193	204	242	246	203	350	385	396	-	> 300	mmHg
A-aDO2	-	579	586	300	294	203	291	253	104	99	85	-	-	mmHg
RI	-	8.3	8,4	2.2	2.1	1.4	1.7	2.1	0.7	0.6	0.4	-	< 0.4	-
HCO3	37.42	35	40.2	33.9	33.2	38.7	32.7	35.1	28.9	27.4	24	-	22 – 28	mmol/L
White Blood Cells	26.79	10.52	-	7.5	-	5.75	-	7.35	-	6.9	-	6.07	3.6 – 10	10^3/µL
Neutrophils	18.52	9.36	-	5.49	-	4.16	-	5.77	-	5.07	-	3.38	1.4 – 6.5	10^3/µL
Eosinophils	0.03	0.01	0.04	0.01	0.00	0.01	0.01	0.00	0.02	0.27	0.04	-	-	10^9^/L
C-reactive protein	63.36	204.4	-	56.8	-	19.1	-	16.3	-	16.3	-	-	<5	mg/L
Creatinine	0.59	0.6	-	0.48	-	0.4	-	0.38	-	0.3	-	0.6	0.57 – 1.11	mg/dL

paCO2 - The partial pressure of carbon dioxide; paO2 - The partial pressure of oxygen; HCO3 - Bicarbonate; P/F Ratio - Horowitz ratio, A-aDO_2_ - Alveolar-arterial oxygen gradient, RI - Respiratory index

In the ICU the patient received the following therapies: protective mechanical ventilation according to the ARDS guidelines associated with prone positioning, empirical antibiotics, fluid resuscitation (crystalloids), anticoagulation (Sodium enoxaparin), vitamin C (*Vitamin C Arena®, Arena Group S.A., Romania*), antipyretics when she exhibited fever (Metamizole, Acetaminophen), mucolytics (Acetylcysteine.), gastric protection with proton pump inhibitors, hepatic protection, loop diuretics like furosemide (*Furosemid Terapia®, Terapia – Sun Pharma, Romania*) and corticosteroids (Methylprednisolone) and nutrition-Nutrison 1kcal/ml (*Nutrison®, Nutricia, Netherlands*).

The monitoring of this patient comprised noninvasive blood pressure, an EKG with 3 leads, SpO2, capnography, a urinary catheter, a nasogastric tube, an arterial line from the right radial artery, and a central venous catheter from the left internal jugular vein.

Despite our maximal therapy, the patient was getting worse, the cultures (blood, bronchial lavage, urine) kept coming negative, and the oxygenation was worsening until the third day of ICU stay, the day when we identified the helminth (Table 1).

**Fig. 2.**
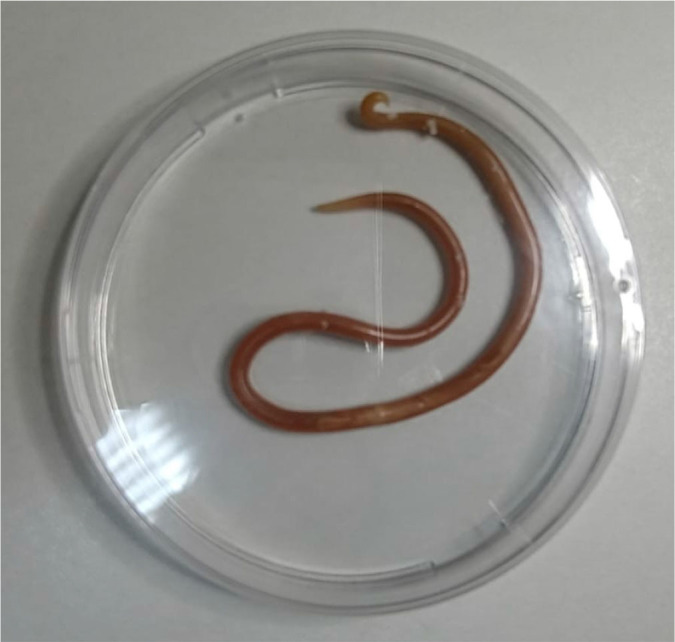
Ascaris lumbricoides extracted with the nasogastric tube

Despite maximal therapy, the patient’s respiratory status continued to deteriorate, with worsening hypoxemia and persistent negative cultures (blood, tracheal aspirate, and urine). On day 3, during routine nasogastric tube replacement, a large adult Ascaris lumbricoides helminth was unexpectedly extracted along with the tube (Figure 2). This critical finding prompted a re-evaluation of the case and initiation of targeted anthelmintic therapy with albendazole (*Zentel®, GlaxoSmithKline, UK*).

The identification of Ascaris lumbricoides shifted the diagnostic focus, and despite initial severe hypoxemia, the patient gradually improved following the introduction of anthelmintic therapy, enabling successful extubation on day 8 and discharge from the ICU on day 12 (Table 2).

**Table 2.** Chronological Patient Monitoring Report – ICU

**Day**	**Urine (mL/day)**	**CVP (cmH_2_O)**	**Cultures**	**GCS**	**RASS**	**ATBx**	**Steroids**	**Sedation / Neuromuscular Blockade**
1	800	15	Blood culture pending; tracheal aspirate collected. Respiratory panel negative – (COVID, Flu, Influenza)	-	−3	Vancomycin Meropenem Moxifloxacin (Avelox®, Bayer, Germania)	Methylprednisolone	Fentanyl + Midazolam
2	1700	17	Cultures: negative	-	−2	Vancomycin + Meropenem + Moxifloxacin	Methylprednisolone	Fentanyl + Midazolam, Rocuronium bromide
3	2400	15	Ascaris Lumbricoides identified	-	−3	Vancomycin + Meropenem + Moxifloxacin + Albendazole	Methylprednisolone	Fentanyl + Midazolam, Rocuronium bromide
4	3000	12	-	-	−2	Vancomycin + Meropenem + Moxifloxacin + Albendazole	Methylprednisolone	Fentanyl + Midazolam, Rocuronium bromide
5	2800	9	-	-	−3	Vancomycin + Meropenem + Moxifloxacin + Albendazole	Methylprednisolone	Fentanyl + Midazolam, Rocuronium bromide
6	4300	13	–	-	−3	Vancomycin + Meropenem + Moxifloxacin + Albendazole	Methylprednisolone Tapered dose	Fentanyl + Midazolam, Rocuronium bromide
7	2400	16	–	7	−3 after sedation restart	Vancomycin + Meropenem (Moxifloxaci stopped)	Methylprednisolone Tapered dose	Sedation interrupted for neurological evaluation; severe agitation & tachypnea; sedation restarted: Fentanyl + Midazolam
8	4700	9	–	15	0	Vancomycin + Meropenem		Sedation stopped (patient extubated)
9	3600	8	–	15	0	Vancomycin + Meropenem	–	–
10	3800	8	–	15	0	Ceftazidime (Fortum®, GSK, UK)	–	–
11	3000	9	–	15	0	Ceftazidime	–	–
12	3500	9	–	15	0	Ceftazidime	–	discharged from ICU

CVP – Central Venous Pressure; GCS- Glasgow Coma Score; RASS – Richmond Agitation Sedation Scale; ATBx- Antibiotics.

Methylprednisolone was initiated at 1 mg/kg/day (≈ 80 mg IV daily) and tapered after day 6.

All drugs were manufactured by the producers and countries mentioned in the text.

**Fig. 3.**
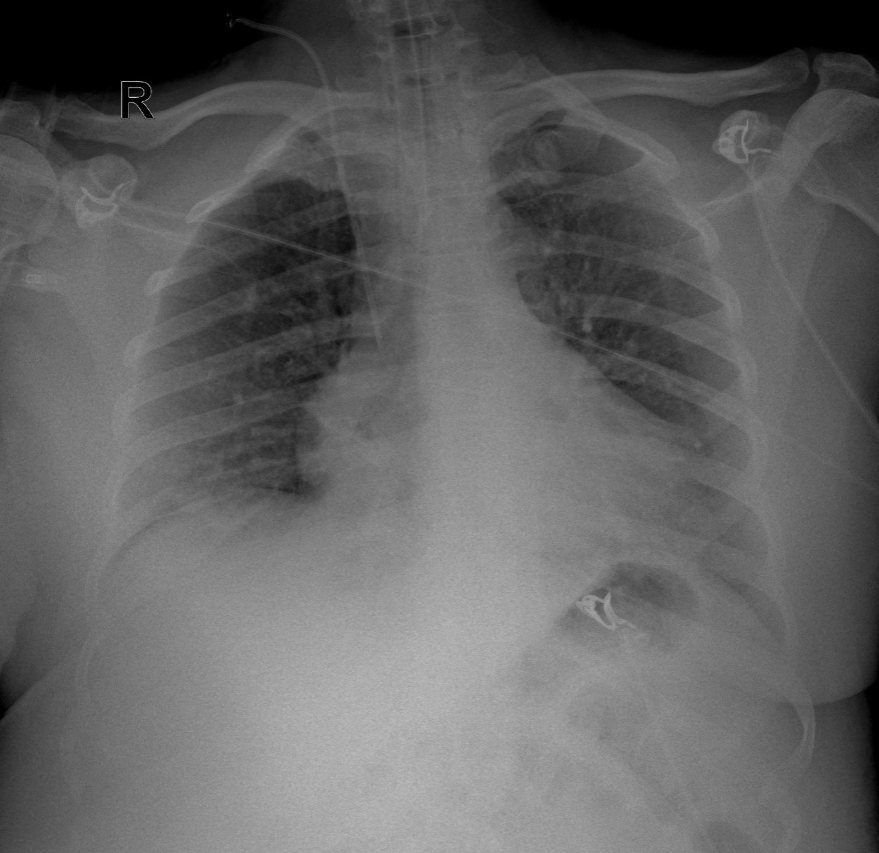
Anteroposterior chest X-ray performed on day 12 of ICU stay (day 12 of ARDS evolution) and day 4 after extubation, showing significant improvement in lung aeration.

On the 12th day of the ICU stay, a pulmonary X-ray was performed, showing significant improvement in the appearance of the lungs (Figure 3).

## Discussion

Ascaris lumbricoides, the largest human intestinal nematode, poses significant health risks, particularly in regions characterized by inadequate sanitation. The global prevalence of Ascaris infections is high, with estimates suggesting around 891 million individuals are affected globally [11]. In addition to gastrointestinal manifestations, the migration of Ascaris larvae through the human body can lead to various pulmonary complications, including Löffler syndrome, which manifests as respiratory symptoms such as cough and may lead to acute respiratory distress syndrome (ARDS) in severe cases [12]. The mechanisms through which Ascaris infection can lead to ARDS are multifactorial. A primary mechanism is the direct migration of larvae into the pulmonary vasculature, resulting in inflammation within the lung parenchyma. This inflammation is characterized by a prominent eosinophilic response, which can lead to symptoms like hypersensitivity pneumonitis and acute lung injury [13]. Elevated eosinophil levels in patients have been shown to correlate with larval migration, resulting in significant pulmonary infiltrates [14,15].

The presence of larvae in the lungs can trigger a Th2-mediated immune response, contributing to eosinophilic pneumonitis and subsequent ARDS [16]. Moreover, Ascaris lumbricoides infections can disrupt the integrity of the gut mucosa, increasing susceptibility to secondary bacterial infections. This systemic inflammation can exacerbate pulmonary injuries, complicating the clinical picture. The interplay between gut pathogens and respiratory health underscores the importance of understanding the implications of systemic helminth infections [17]. In severe cases of Ascaris infections, the detection of adult worms in the gastrointestinal tract, alongside respiratory symptoms, offers crucial evidence linking helminth-induced ARDS, emphasizing the need for heightened surveillance for such cases, particularly in endemic areas [18].

In temperate regions such as Romania, Ascaris lumbricoides is not typically considered a major respiratory pathogen. However, localized endemicity persists, particularly in rural and socioeconomically disadvantaged areas with inadequate sanitation. Epidemiological analyses indicate that ascarid infections remain present, especially among children. For example, a global review by Jourdan et al. reports that despite deworming programs, Romania continues to report cases of ascariasis, with notable prevalence in some regions [11]. These data reinforce the importance of maintaining a broad differential diagnosis, even in non-tropical settings, when evaluating patients with unexplained ARDS.

Although a standard diagnostic protocol was not defined, we now emphasize the potential role of adjunctive investigations such as eosinophil count (which was monitored daily in our patient and remained within normal limits), stool examination (which confirmed the diagnosis post-discovery), and chest CT imaging (which was nonspecific in this case). Serological testing was not performed due to its known limitations, including cross-reactivity and difficulty in distinguishing acute from chronic infection.

In the presented case, the absence of detectable bacterial or viral pathogens, along with severe hypoxemia and bilateral infiltrates, supports the diagnosis of helminth-induced ARDS. Although rare, this condition represents a significant clinical entity, especially in high-risk populations exposed to environmental factors [20, 21]. Recognizing such conditions is essential for improving management strategies and outcomes for affected individuals in endemic regions. Moreover, it can be a look outside the box, when nothing else checks out, think about pathogens not often encountered in a critically ill patient.

To our knowledge, no previous case of ARDS induced by Ascaris lumbricoides infection has been published in Romania, despite the parasite’s continued presence in certain rural areas. Although our diagnosis was established indirectly—through exclusion of viral, bacterial, and other common causes—the clinical evolution, imaging, and identification of the parasite support a causative role. Notably, peripheral eosinophilia, which is typically expected in parasitic infections, was absent throughout the ICU stay, highlighting the potential for atypical presentations and diagnostic uncertainty. This case underscores the importance of considering parasitic etiologies in young patients with unexplained ARDS, even in the absence of traditional markers or risk factors. In such contexts, maintaining a broad differential diagnosis is essential for timely recognition and targeted therapy.

In conclusion, Ascaris lumbricoides poses risks to gastrointestinal health and respiratory functions, culminating in conditions like ARDS. Understanding the immunological responses and potential pathways leading to these severe complications underscores the necessity of addressing helminth infections in clinical assessments and public health initiatives.

Written informed consent was obtained from the patient for publication of this case report and accompanying images, Ethics Committee approval number 4640/2025
